# Fifth Anniversary of *Rambam Maimonides Medical Journal*: From Concept to Action and Success

**DOI:** 10.5041/RMMJ.10207

**Published:** 2015-07-30

**Authors:** Shraga Blazer, Karl L. Skorecki, Rafael Beyar

**Affiliations:** 1 Editor-in-Chief, Rambam Maimonides Medical Journal, Rambam Health Care Campus, Haifa, Israel; 2 Director of the Department of Neonatology and Neonatal Intensive Care Unit, Rambam Health Care Campus, Haifa, Israel; 3 Ruth & Bruce Rappaport Faculty of Medicine, Technion – Israel Institute of Technology, Haifa, Israel; 4 Associate Editor, Rambam Maimonides Medical Journal, Rambam Health Care Campus, Haifa, Israel; 5 Director of Medical and Research Development, Rambam Health Care Campus, Haifa, Israel; 6 General Director, Rambam Health Care Campus, Haifa, Israel.

Dear Friends and Colleagues,

An anniversary is not only a point of memory—it provides the opportunity for self-examination and paves the way to the future. Every anniversary marks a starting-point that was preceded by a vision. The beginning of any vision is a personal dream—someone wants to improve or repair the world as far as he is able. The vision motivates action; in its aftermath comes the reality. This is the 21st issue of *Rambam Maimonides Medical Journal*. This issue is particularly important as it marks the completion of five years of creative work pursuing our vision for a high-caliber scientific medical journal. Our vision has become reality.

The impact a physician has in the lives of patients is far more than physical—it is emotional, intellectual, and sometimes even spiritual. Nevertheless, that influence rarely extends beyond the individual patient, although in some cases it may reach their extended family. Yet knowledge regarding medical and scientific research can deeply impact our society if that information is made available and accessible to the entire medical community as well as the greater public. This concept stands behind our motivation to initiate and sponsor *Rambam Maimonides Medical Journal*.

Several key concepts were established at the onset of our “Maimonides” journey. First and foremost, we envisioned an international, multidisciplinary, open-access (free to both our writers *and* our readers), peer-reviewed publication. We envisioned top contributors in their respective fields who would bring together their scientific expertise combined with their personal stance and experience.

In developing an editorial policy, we were guided by the highest standards of scientific quality and integrity, professional responsibility, and human compassion in accordance with the Rambam’s (Maimonides) scholarly and ethical legacy. Maimonides’ monumental work on Jewish law, ethics, and medical practice are classic and remain remarkably relevant today. Rambam Health Care Campus proudly bears the Hebrew name of Maimonides and aspires to be guided by his principles. Hence the name of our new publication was quickly decided upon: *Rambam Maimonides Medical Journal*. In the spirit of the Maimonides’ quest for integrity in research and dissemination of truth, the journal’s mission was defined: to expand the knowledge base of medicine, science, humanity and ethics throughout the world, flavored by the salt of the 800-year-old philosophy of Rabbi Moshe Ben Maimon—the Maimonides.

We had to overcome technical challenges with limited resources. We developed a proprietary computer system to specifically meet the journal’s needs. Today, every step of the publication process, from submission to release, is fully computerized. We are now able to provide our readers with downloadable PDFs, the ability to read online in a browser, and articles can now be accompanied by videos.

Based on the data, we have every reason to be proud—we have succeeded in our goal. Many of our writers are world leaders in medicine, ethics, humanity, and science. Among them are eight Nobel Laureates. Published articles focus on a wide spectrum of clinically relevant biomedical research and other issues central to the provision of optimal health care, disease prevention and environmental health, Jewish and general ethics, philosophy and the history of medicine, and much more. Content even includes transcripts of public debates, and there have been several issues dedicated to specific topics of interest.

Over the past five years, *Rambam Maimonides Medical Journal* has achieved outstanding attention, demonstrated by more than 15,900 registered readers, with more than 262,000 page views by readers from 158 countries and territories. The journal’s quality is internationally recognized: *Rambam Maimonides Medical Journal* is a member of Cross Ref and is indexed by PubMed, EBSCO, and Google Scholar—to name a few.

We have not been alone in the realization of this endeavor. We are grateful to all of our families, colleagues, and collaborators who have supported and encouraged us along the way. We are deeply grateful to our Editorial Board—they have supported us throughout our Maimonides journey and encouraged us to forge ahead when very few believed in the journal’s potential for success.

We are thankful for each contributor who has provided us with outstanding work, and to the anonymous reviewers who have faithfully undertaken honest and in-depth critiques for the benefit of the scientific and medical community. Your contributions have assured that our loyal readership finds something of interest in every issue.

We would like to extend special thanks to two individuals who are the heart and soul of our editorial office, our Editorial Assistant, Mrs. Deborah Hemstreet, and our gifted and committed copy editor, Mrs. Lisbet Clements. Almost every one of our writers, at some point during manuscript processing, has written to thank our office for their meticulous, professional, and good-natured help. You, our readers, have benefited from their professionalism.

*Rambam Maimonides Medical Journal* was founded as an independent and free journal, not supported by any organization or medical society. To date, the generosity of donors has enabled the journal to remain freely accessible via the internet for immediate worldwide open access to the full text of articles. The journal fulfills the terms of the Creative Commons attribution license under which everyone is free to copy, distribute, and display the work, free of charge for non-commercial personal and educational purposes. We particularly want to thank the Friends of Rambam (Israel), our donors and friends in Israel and worldwide—most of whom have asked to remain anonymous—for their generous support.

Finally, we would like to thank you, our faithful readers. You are the final word of our success. Every download, every page accessed by you, is yet another indication that our vision has been realized. We aim to continue publishing high-quality papers written by esteemed interdisciplinary scholars. As you continue to enjoy the benefits achieved from reading *Rambam Maimonides Medical Journal*, we also welcome you to contribute from your expertise and experience. We are also pleased to receive Letters to the Editor regarding previously published articles. Join us in the Maimonides journey!

Please consider making a contribution to assure that our journal continues to remain advertising-free, and free to everyone. Be sure to spread the word about this unique publication, and don’t forget to cite articles published in *Rambam Maimonides Medical Journal*. Our new vision is to further our acceptance and scientific strength as proof of the importance of freely accessible knowledge and honoring to the Rambam.

This Fifth Anniversary issue was written with you in mind. Members of our Editorial Board, and Sir John Gurdon, an emeritus board member and Nobel Laureate, have contributed of their broad expertise on a variety of topics.

Thank you for being a part of the past and the future of *Rambam Maimonides Medical Journal*.

